# French Vividness of Olfactory Imagery Questionnaire: A Potential Tool for Diagnosing Olfactory Loss by Assessing Olfactory Imagery?

**DOI:** 10.3389/fpsyg.2020.606667

**Published:** 2020-12-21

**Authors:** Luca Fantin, Hadrien Ceyte, Zhor Ramdane-Cherif, Muriel Jacquot, Gabriela Hossu

**Affiliations:** ^1^Université de Lorraine, INSERM, IADI, Nancy, France; ^2^Université de Lorraine, DevAH, Nancy, France; ^3^CIC 1433 Innovation Technologique, INSERM, Université de Lorraine, CHRU Nancy, Nancy, France; ^4^Université de Lorraine, InnoCIM, ENSAIA, Myrissi, Nancy, France

**Keywords:** olfactory imagery, olfactory ability, smell loss, anosmic, odor expert, normosmic, French questionnaire

## Abstract

Several studies have shown a significant relationship between smelling and olfactory imagery abilities. The primary aim of the present study was to validate a French version of the Vividness of Olfactory Imagery Questionnaire (VOIQ). The secondary aim was to investigate its capability to differentiate individuals with smell loss from healthy individuals. After having elaborated a French translation of the VOIQ (fVOIQ), we evaluated olfactory imagery abilities of 387 French participants who anonymously self-completed the fVOIQ: 121 pathologic individuals (hyposmic and anosmic), 244 normosmic individuals (healthy non-expert), and 22 fragrance experts. Significant split-half reliability as expressed by Spearman correlation coefficients for the global sample, as well as for each group separately, indicated the excellent internal consistency of the fVOIQ. Moreover, results revealed a significant effect of the smelling ability group on fVOIQ score, suggesting that daily olfactory stimulation is fundamental to maintaining the ability to create a vivid image and that severe loss of smell may result in progressive impairment of olfactory imagery. Our fVOIQ and the original English version seemingly have similarly high benefit in differentiating experts and normosmic individuals based on their olfactory imagery ability. Moreover, the fVOIQ seems capable of differentiating individuals with loss of smell from healthy individuals. These findings demonstrate the reliability and validity of the fVOIQ, and its capability to differentiate individuals’ smelling ability according to their olfactory imagery ability.

## Introduction

Sensory imagery refers to the volitional mental simulation of sensory experiences and is known to be feasible in every sensory modality (Andrade et al., 2012). Several studies have shown that recall of sensory information is possible thanks to modality-specific neural networks, which overlap with those of their respective sensory modalities (Farah, 1988; Kosslyn et al., 2001). Consequently, there are forms of coherence between real perception and sensory imagery. For example, consistencies can be observed between perceived and imaged stimuli. In the case of olfaction, the pleasantness and intensity of a smell are preserved when imaged (Carrasco and Ridout, 1993; Sugiyama et al., 2006). However, of all senses, olfactory imagery seems to be particularly challenging (Brower, 1947; Lindauer, 1969). The lesser importance of olfaction in everyday life and the unawareness of having formed an image are potential reasons for the difficulty of olfactory imagery (Stevenson and Case, 2005). Therefore, wider interindividual variability can be observed during olfactory imagery tasks, than in any other sensory modality.

Numerous factors influencing olfactory imagery vividness have been studied. Flohr et al. (2014) studied the effect of smell loss on the ability to create an image. This study demonstrated that olfactory loss was an important factor in olfactory imagery ability. Regular exposure to olfactive stimuli would be key in forming an image. Therefore, many pathologies resulting in smell loss could impact imagery, such as sinonasal polyposis, or coronavirus (Lechien et al., 2020). Therefore, the assessment of olfactory imagery ability in regard to its relationship with real odor perception could provide help for the early assessment of smell loss. A specific tool capable of assessing olfactory imagery ability is thus needed.

Several authors have worked on creating questionnaires assessing sensorial imagery (Betts, 1910; Sheehan, 1967; Marks, 1973; White et al., 1978), though few specifically assess olfactory imagery. Questionnaires based on Betts’ Questionnaire upon Mental Imagery, which was initially developed for assessing 150 items pertaining to all sensory modalities, are time-consuming, which can induce a bias when they are completed. Gilbert et al. (1998) developed the Vividness of Olfactory Imagery Questionnaire (VOIQ) adapted from the Vividness of Visual Imagery Questionnaire (VVIQ—Marks, 1973). Specific to the assessment of olfactory imagery, the VOIQ offers a contextual approach to the odors that are to be imagined. The total of 16 items makes the VOIQ a reasonably short questionnaire, which can be completed within 5–10 min. Its simplicity makes it possible for individuals to complete on their own. Gilbert et al. (1998) showed that the VOIQ allows differentiation between the olfactive imagery abilities of fragrance experts and healthy non-expert individuals. Therefore, the regular exposure to olfactory stimuli may be a key to form a vivid image (Royet et al., 2013). Moreover, Flohr et al. (2014) including 16 patients with severe smell loss suggested that olfactory loss might be an important factor in imagery abilities. However, these findings need to be confirmed with larger samples. Currently, the VOIQ is adapted to English-speaking populations. Therefore, the development of a French version of this questionnaire is of great interest.

The primary aim of the present study was to validate a French version of the VOIQ (fVOIQ). The secondary aim was to investigate its capability to differentiate individuals with smell loss from healthy individuals. To accomplish this, we compared olfactory imagery abilities of fragrance expert, normosmic (healthy non-expert), and pathologic (hyposmic and anosmic) individual samples. We hypothesized that experts would have a better olfactory imagery ability as compared to healthy non-expert and pathologic individuals. Similarly, we expected better olfactory imagery ability for healthy non-expert individuals as compared to those who were pathologic.

## Materials and Methods

### Participants

387 participants volunteered for this study (see Table 1). They were recruited by either a web-based (web) or a paper & pencil (p&p)-administered questionnaire. All participants were asked to anonymously self-report their gender, age group, and to categorize their ability to perceive smells orthonasally *a priori* into one of four groups: (1) anosmic (either born unable to smell—congenital anosmic—or having developed the inability), (2) hyposmic, (3) normosmic, and (4) expert (i.e., professional fragrance experts). All participants who were recruited by the paper & pencil questionnaire declared themselves as normosmic. Each participant was then classified (according to their declaration) into one of four groups: one pathologic group (*n* = 121), which included hyposmic and anosmic participants, two normosmic groups (*n*_*p*__&__*p*_ = 21 and *n*_*web*_ = 223), and one expert group (n = 22).

**TABLE 1:** Participants’ characteristics according to questionnaire completion condition: web-based or paper and pencil.

		**Web-based**	**Paper & pencil**
Gender		300 female–66 male	21 female–0 male
Age interval	[18;25]	121 (33%)	21 (100%)
	[26;45]	91 (25%)	0
	[46;54]	59 (16%)	0
	[55; +]	95 (26%)	0
Orthonasal smell perception ability	Anosmic	108 (30%, 17 congenital)	0
	Hyposmic	13 (3%)	0
	Normosmic	223 (61%)	21 (100%)
	Expert	22 (6%)	0
Global		366 (100%)	21 (100%)

### Instrument

The original English version of the VOIQ was first transcribed into French with the help of collaborators fluent in English and native in French. The translation was then presented to a professional translator, fluent in French, and native in English, who back translated the questionnaire to English and made corrections by comparing it to the original questionnaire. In addition, French healthy internal collaborators were also asked to note whether the questionnaire translation was clearly understandable. If it was not, they had to specify which item was not understood. Thus, amendments to the French version were progressively made until both parties fully agreed on the final translated document.

### Procedure

In the manner of the original version of VOIQ, the fVOIQ is composed of 16 items split into 4 situations for which 4 specific smells are presented (see Table 2). Each participant was instructed to read each sentence and imagine the considered smell. They were then asked to rate the vividness of the imagined smell on a scale from 1 to 5 where each score corresponds to the rating scale presented in Table 2.

**TABLE 2:** VOIQ and fVOIQ rating scales and 16 items of questionnaires split into four situations for which four specific smells.

**VOIQ**		**fVOIQ**

	RATING SCALE	

**∙** Perfectly realistic and as vivid as the actual odor	**Rating 1**	**∙** Parfaitement réaliste et aussi vive que la véritable odeur
**∙** Realistic and reasonably vivid	**Rating 2**	**∙** Réaliste et raisonnablement vive
**∙** Moderately realistic and vivid	**Rating 3**	**∙** Modérément réaliste et vive
**∙** Vague and dim	**Rating 4**	**∙** Vague et terne
**∙** No odor at all, you only “know” that you are thinking of the odor	**Rating 5**	**∙** Pas d’odeur du tout, vous “savez” simplement que vous pensez à une odeur

	ITEMS	

**Think of a time when you really need to take a bath or shower—your clothes are smelly and you need to wash your hair.**		**Pensez à un moment où vous avez vraiment besoin de prendre un bain ou une douche—vos vêtements sentent mauvais et vous devez vous laver les cheveux.**
1. The smell of your shirt or blouse when you remove it.		1. L’odeur de votre chemise ou chemisier lorsque vous l’enlevez.
2. The fragrance of the soap or shampoo you use to wash.		2. Le parfum du savon ou du shampooing que vous utilisez pour vous laver.
3. The smell of the fresh clothes you put on.		3. L’odeur des vêtements propres que vous enfilez.
4. The odor of an aftershave, perfume, or cologne you use afterward.		4. L’odeur de l’après rasage, du parfum ou de l’eau de toilette que vous utilisez après.
**Think of an outdoor cookout or barbeque. Consider the smells that occur.**		**Pensez à un barbecue et aux odeurs présentes.**
5. The charcoal or wood has just been lit and is beginning to burn.		5. Le charbon ou le bois vient d’être allumé et commence à brûler.
6. The food has been cooking on the grill and is almost done.		6. La nourriture cuit sur le gril et est bientôt prête.
7. The smell of the food as you savor the first bite.		7. L’odeur de la nourriture pendant que vous savourez la première bouchée.
8. The stench as leftover garbage is burned on the fire.		8. La puanteur des déchets restants qui brûlent sur le feu.
**Think of someone you know who smokes tobacco. Bring to mind the smells associated with it.**		**Pensez à quelqu’un que vous connaissez qui fume du tabac. Rappelez-vous des odeurs qui y sont associées.**
9. The odor of unlit tobacco—a cigarette, cigar or pouch of pipe tobacco.		9. L’odeur des feuilles de tabac—une cigarette, un cigare, ou du tabac à rouler.
10. A dense cloud of tobacco smoke fills the room.		10. Un nuage dense de fumée de cigarette remplit la pièce.
11. The odor of slate cigarette or cigar butts in an ashtray.		11. L’odeur du tabac froid des mégots dans un cendrier.
12. The lingering smell of tobacco smoke on your cloths after you leave the room.		12. L’odeur persistante de la fumée de tabac sur vos vêtements après avoir quitté la pièce.
**Finally, think of a familiar car, and getting into it and going for a ride.**		**Finalement, pensez à une voiture familière, à vous installer dedans et à conduire.**
13. The odor inside the car—the upholstery and other items.		13. L’odeur à l’intérieur de la voiture—le cuir/plastique et les autres éléments.
14. The smell of exhaust from a passing truck.		14. L’odeur de l’échappement d’un camion qui vous dépasse.
15. You smell gasoline as the tank is being filled.		15. L’odeur de l’essence pendant que vous faites le plein.
16. Inside a service station—the smell of new rubber tires and grease.		16. Dans une station-service—l’odeur de nouveaux pneus et de la graisse.

As previously indicated, either the web or pen and paper version of the fVOIQ was self-administered. Regardless of the form of administration, participants were not asked to provide any identifying data.

### Data Analysis

Raw data for each participant (gender, age interval, orthonasal smell perception ability, and fVOIQ global score) were stored in a specific database. The mean fVOIQ score was calculated for each participant (sum of answers/16 items). A low mean score (close to 1) indicates a good ability to imagine smells, and vice versa.

All statistical analyses were performed using R version 3.6.3. The threshold for statistical significance was α = 0.05. fVOIQ score distribution for all four groups differed from the normal distribution (Shapiro–Wilk test, *ps* < 0.001). Therefore, quantitative data were expressed as medians (Med) associated with interquartile range (IQR) and qualitative data by their frequencies.

In order to verify the reliability of the fVOIQ, the internal consistency of the questionnaire was assessed. Split-half reliability of the fVOIQ was tested by Spearman correlations between average scores on odd items and even items for each participant. The Mann–Whitney U test was used to compare fVOIQ scores between groups (web-based or paper & pencil administered questionnaire). A Kruskal–Wallis non-parametric ANOVA was used to study group effect (pathologic, normosmic and expert) on olfactory imagery ability, i.e., their median fVOIQ score. Tests were adjusted for multiple comparisons according to Bonferroni–Holm correction.

## Results

There were no significant differences in fVOIQ scores between web-based and paper & pencil conditions (Med_*p*__&__*p*_ = 2.69 ± 0.69; Med_*web*_ = 2.31 ± 1.25; *Z* = −1.09; *p* > 0.05). Therefore, all normosmic participants were grouped for further analyses, reducing the number of groups to three: one pathologic group (*n* = 121), one normosmic group (*n* = 244), and one expert group (*n* = 22).

### Internal Reliability of the fVOIQ

**FIGURE 1:**
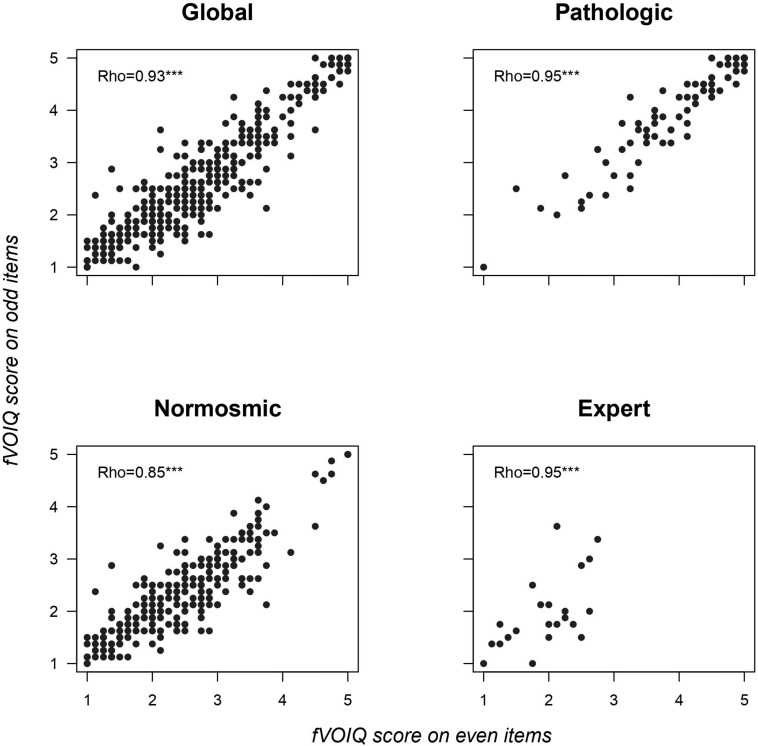
Split-half reliability expressed by Spearman correlation coefficients for global population and each group (pathologic, normosmic, and expert). ***p < 0.001.

Split-half reliability as expressed by Spearman correlation coefficients was statistically significant for the global sample, as well as for each group separately (Figure 1), thus verifying the internal consistency of the fVOIQ.

### Olfactory Ability Differentiation by fVOIQ Score

**FIGURE 2:**
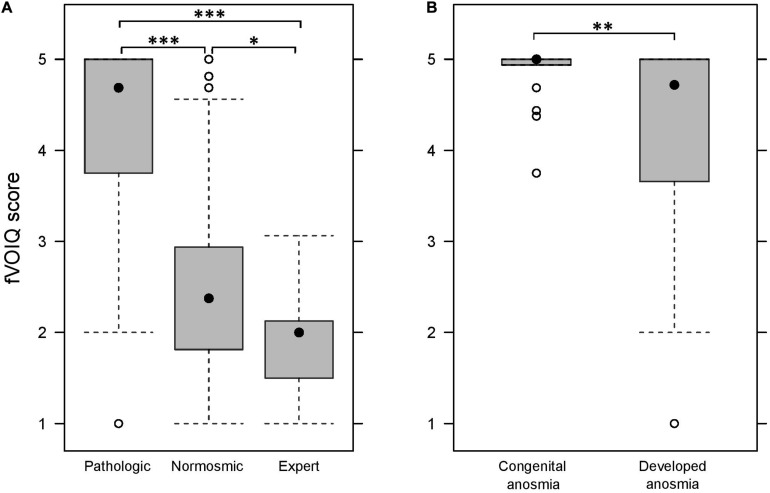
fVOIQ scores in each group, i.e., pathologic, normosmic, and expert **(A)** and for each type of anosmia **(B)**. *p < 0.05; **p < 0.01; ***p < 0.001.

The Kruskal–Wallis non-parametric ANOVA showed a significant effect of group on the median fVOIQ score [H(2, *N* = 387) = 211.6; *p* < 0.001]. As illustrated in Figure 2A, the score for the pathologic group (Med_*pat*_ = 4.69; IQR = 1.25) was significantly higher than both normosmic and expert groups (Med_*nor*_ = 2.38, IQR = 1.13 and Med_*exp*_ = 2.00, IQR = 0.63; corrected *ps* < 0.001). The score for the normosmic group was also significantly higher than that of the expert group (corrected *p* < 0.05).

The differentiation power of the fVOIQ was further tested in the pathologic group, by comparing congenital anosmic participants, to those who had developed anosmia (Figure 2B). Comparison was performed with a Mann–Whitney U test for independent samples. Results revealed that congenital anosmic participants (Med = 5.00; IQR = 0.06) had significantly higher fVOIQ scores than participants with developed anosmia (Med = 4.69; IQR = 1.38; *Z* = −2.79; *p* < 0.01).

## Discussion

The primary aim of this study was to validate a questionnaire allowing to assess olfactory imagery ability in French-speakers. The internal consistency of our fVOIQ was high on the global population, and in each group. In line with our hypothesis, the fVOIQ corroborated previous findings using Gilbert et al.’s (1998) original English version, that is to say, better olfactory imagery ability in fragrance experts, than normosmic individuals. These findings suggest that regular exposure to strong olfactory stimuli is a key to form an olfactory representation (Bensafi and Rouby, 2007; Royet et al., 2013). This was supported by the results of the fragrance experts in our study, although this effect could be underestimated by a recruiting effect in normosmic volunteers as suggested by Arshamian et al. (2011). Indeed, these authors proposed that this recruiting method may have appealed to individuals with high odor interest, and therefore higher olfactory imagery abilities. Therefore, this study has demonstrated that the fVOIQ is reliable and a valid tool for assessing olfactory imagery ability in French populations.

Our results showed a wide variation in normosmic individuals, allowing us to distinguish good odor imagers, from poorer imagers (Bensafi et al., 2005). In addition to this, and despite the differences in the number of participants in each group, our findings suggest a potential benefit of the fVOIQ in discriminating individuals with smell impairments. Indeed, participants with loss of smell had significantly higher fVOIQ scores than fragrance experts and healthy non-experts, meaning they had more difficulty imagining odors. Therefore, more so than regular strong odor exposure, natural and daily olfactory stimulation is fundamental to maintaining the ability to create a vivid image. The relationship between smelling and olfactory imagery abilities is thus further substantiated. Moreover, the significant difference between congenital anosmic and participants with developed anosmia reveals that the fVOIQ provides the ability to differentiate these two populations. This finding suggests that severe loss of smell results in progressive impairment of olfactory imagery ability. Further studies are needed to determine whether the fVOIQ is a consistent tool to evaluate the severity level of olfactory impairment in individuals with smell loss.

Lastly, it is important to note that contextualized olfactory imagery vividness depends on culture (Stevenson and Boakes, 2003), which is why all participants in this study were French. However, the questionnaire used in this study may not apply to all French-speaking populations, as some items or situations could not be relatable to individuals of certain countries with other cultures.

A perspective of application of the fVOIQ could also be the diagnosing of olfactory impairments. Indeed, according to the European Rhinologic Society, patients today with a sudden loss of smell should be considered as potentially COVID-19 positive. A significant proportion of COVID-19 patients (20–60%) experience loss of smell or taste (Mazzatenta et al., 2020). Recent findings suggest that loss of smell as a result of COVID-19 could be caused by alteration of the posterior gyrus rectus and olfactory bulbs (Politi et al., 2020). This symptom can predict infection to this coronavirus (Lechien et al., 2020), as it appears prior to other symptoms such as coughing or fever. In this context, measuring one’s olfactory ability could be a way of diagnosing COVID-19 early in many individuals. However, contagion risks are high, and protective medical equipment and detection procedures are time intensive and costly. The present study allows us to suggest that the assessment of one’s olfactory imagery abilities could provide a self-administered preliminary diagnosis of COVID-19, maintaining social distancing and limiting financial cost. Likewise, the original VOIQ for English-speaking populations (representing over 2 billion people) could be a relevant tool as it is quick, free, non-invasive, and self-administered (independently of the administration method) and safe in the COVID-19 context.

## Conclusion

The fVOIQ appears to have high reliability and validity, when compared to the data of the original English VOIQ. It can therefore be used in a French population for assessing olfactory imagery ability and could also differentiate individuals with smell loss from healthy individuals.

## Data Availability Statement

The raw data supporting the conclusions of this article will be made available by the authors, without undue reservation, to any qualified researcher.

## Ethics Statement

Ethical review and approval was not required for the study on human participants in accordance with the local legislation and institutional requirements. Written informed consent for participation was not required for this study in accordance with the national legislation and the institutional requirements.

## Author Contributions

GH and MJ conceived, designed the study, conducted the experiments, collected, and processed the data. LF, GH, and HC analyzed the data. LF, HC, MJ, and GH wrote the manuscript. All authors contributed to the article and approved the submitted version.

## Conflict of Interest

The authors declare that the research was conducted in the absence of any commercial or financial relationships that could be construed as a potential conflict of interest.
